# Genome sequencing and description of *Oerskovia enterophila* VJag, an agar- and cellulose-degrading bacterium

**DOI:** 10.1186/s40793-017-0244-4

**Published:** 2017-05-04

**Authors:** Vanessa Jag, Anja Poehlein, Frank R. Bengelsdorf, Rolf Daniel, Peter Dürre

**Affiliations:** 1 Institut für Mikrobiologie und Biotechnologie, Universität Ulm, Albert-Einstein-Allee 11, D-89081 Ulm, Germany; 2 Genomic and Applied Microbiology & Göttingen Genomics Laboratory, Institute of Microbiology and Genetics, Georg-August-University Göttingen, Grisebachstr. 8, D-37077 Göttingen, Germany

**Keywords:** *Oerskovia*, *Cellulomonadaceae*, Cellulose degradation, Soil bacteria, Phylogenetic analysis

## Abstract

A nonmotile, Gram-positive bacterium that shows an elongated and branching cell shape was isolated from soil samples from the botanical garden of Ulm University, Ulm, Germany. Here, the isolation procedure, identification, genome sequencing and metabolic features of the strain are described. Phylogenetic analysis allowed to identify the isolated strain as *Oerskovia enterophila.* The genus *Oerskovia* belongs to the family *Cellulomonadaceae* within the order *Actinomycetales*. The length of cells of *O. enterophila* ranges from 1 μm to 15 μm, depending on the growth phase. In the exponential growth phase, cells show an elongated and branching shape, whereas cells break up to round or coccoid elements in the stationary growth phase. The 4,535,074 bp long genome consists of 85 contigs with 3918 protein-coding genes and 57 RNA genes. The isolated strain was shown to degrade numerous complex carbon sources such as cellulose, chitin, and starch, which can be found ubiquitously in nature. Moreover, analysis of the genomic sequence revealed the genetic potential to degrade these compounds.

**Electronic supplementary material:**

The online version of this article (doi:10.1186/s40793-017-0244-4) contains supplementary material, which is available to authorized users.

## Introduction

*Oerskovia enterophila* was formerly characterized as *Promicromonospora enterophila* by Jàger et al. in 1983 [1]. Later, *P. enterophila* was re-classified as *O. enterophila* by Stackebrandt et al. [2], since only spore-like elements and no real spores are formed. Furthermore, a phylogenetic tree based on the 16S rRNA gene sequences of strains of the genera *Cellulomonas* and *Promicromonospora* shows that *O. enterophila* did not cluster with the type species of *Promicromonospora* *,* *Promicromonospora citrea*, or *Promicromonospora sukumoe* [2, 3]. The genus *Oerskovia* was initially described in 1970 by Prauser et al. [4] and harbors currently four species with *O. turbata* as type species [2]. Bacteria of the genus *Oerskovia* belong to the phylum *Actinobacteria*, which is one of the largest taxonomic units among the domain *Bacteria* [5]. Bacteria belonging to *Actinobacteria* show a wide range of G + C-content, from 51% to more than 70% [5–7]. *Actinobacteria* are widely distributed in terrestrial as well as in aquatic habitats [8, 9]. In general, members of the class *Actinobacteria* show a high morphological variety, which is also true for species of the genera *Oerskovia* and *Cellulomonas* [10]. Furthermore, members of the family *Cellulomonadaceae* are known for their ability to decompose plant-derived biopolymers such as starch, cellulose or chitin [11]. Due to the close relationship of members of the genera *Oerskovia* and *Cellulomonas* [12, 13] it is likely that both share genetic features enabling them to degrade these biopolymers. To investigate the genetic potential for biopolymer degradation, the genome of the isolate was sequenced. Furthermore, a genome wide comparison of the isolated strain with other *Oerskovia* type strains was performed. Additionally, the isolated strain was aerobically grown on respective carbon sources to validate the functionality of the proposed degradation pathways.

In this contribution, the classification, the metabolic features, and the genome insights of the isolated strain are provided.

## Organism information

### Classification and features

The isolated strains were identified as *Oerskovia enterophila* based on 16S rRNA gene sequence identities of more than 99% compared to the type strain of *O. enterophila* DSM 43852 [14]. All subsequent analyses were performed using the strain designated as *O. enterophila* VJag. Information regarding the enrichment and isolation procedures as well as identification of *Oerskovia* strains are described in the Additional files 1 and 2: S1 and S2.

**Fig. 1:**
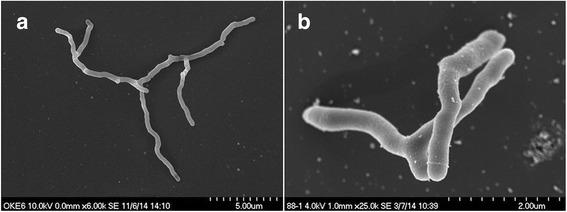
Electron micrograph of *O. enterophila* VJag, using a Hitachi S-5200 scanning electron microscope. **a**: cells from the exponential growth phase; scale bar: 5 μm. **b**: cells from the stationary growth phase; scale bar: 2 μm. Zentrale Einrichtung Elektronenmikroskopie, Universität Ulm

Investigations of the cell morphology of the isolated strain *O. enterophila* VJag (Table 1) using scanning electron microscopy revealed that cells show different morphologies in exponential and stationary growth stage. In the exponential growth phase, cells show extensive branches with an overall length up to 15 μm, whereas the cells are smaller and less branched in the stationary growth phase (Fig. 1). These different cell morphologies were also previously observed by Stackebrandt et al. [2].

**Table 1:** Classification and general features of *O. enterophila* VJag according to the MIGS recommendations [26]

MIGS ID	Property	Term	Evidence code^a^
	Classification	Domain: Bacteria	TAS [39]
		Phylum: *‘Actinobacteria’*	TAS [5]
		Class: *Actinobacteria*	TAS [12]
		Order: *Actinomycetales*	TAS [40–42]
		Family: *Cellulomonadaceae*	TAS [11, 19]
		Genus: *Oerskovia*	TAS [4]
		Species: *Oerskovia enterophila*	TAS [1, 2]
		Strain: VJag (LRIE00000000)	TAS [5, 14]
	Gram stain	Positive	IDA, TAS [4]
	Cell shape	Rods	IDA, TAS [4]
	Motility	Non-motile	IDA, TAS [4]
	Sporulation	Non-sporulating	IDA, TAS [2]
	Temperature range	Mesophile	IDA, TAS [4]
	Optimum temperature	28–30 °C	IDA, TAS [4]
	pH range, optimum	3–11, 7	TAS [1], IDA
	Carbon source	glucose, fructose, mannose, galactose, ribose, xylose, cellobiose, maltose, trehalose, saccharose, lactose	IDA, TAS [1, 2, 4]
MIGS-6	Habitat	Affiliated to gut environments of invertebrates, soil	TAS [11]
MIGS-6.3	Salinity	5–7% (w/v)	TAS [1]
MIGS-22	Oxygen-requirement	Facultative anaerobe	TAS [4]
MIGS-15	Biotic relationship	Free-living, commensal	IDA, TAS [11]
MIGS-14	Pathogenicity	-	
MIGS-4	Geographic location	Botanical garden of Ulm University, Ulm, Germany	IDA
MIGS-5	Sample collection	January 2013	IDA
MIGS-4.1	Latitude	48.42218 °N	IDA
MIGS-4.2	Longitude	9.95922 °E	IDA
MIGS-4.4	Altitude	-	

^a^Evidence code - IDA: Inferred from Direct Assay; TAS: Traceable Author Statement; These evidence codes are from the Gene Ontology project [43]. If the evidence is IDA, then the property was directly observed for a live isolate by one of the authors

The 16S rRNA gene sequence (OJAG_11220, LRIE01000058.1) of *O. enterophila* VJag was blasted [15] and used for subsequent phylogenetic analysis. Therefore, 16S rRNA reference sequences of 17 closely related type strains were aligned using MAFFT version 7.215 [16, 17] and was performed using EMBL-EBI web services. The length of the 17 references ranged from 1395 to 1612 bp and had average length of 1486 bp. The phylogenetic tree was reconstructed using the software MrBayes version 3.2.6 [18]. The recommended settings in the manual for tree reconstruction use a generalized time reversible evolutionary model. The quick start instructions were followed to run Bayesian phylogenetic analysis. The run was stopped since the standard deviation of split frequencies was below 0.0042 after 1,000,000 generations.

**Fig. 2:**
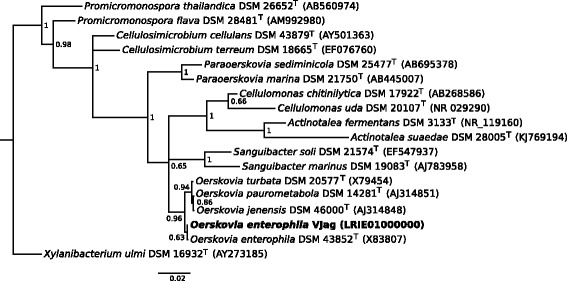
Phylogenetic tree based on the 16S rRNA sequences indicating the genetic relationships between the isolate *O. enterophila* VJag and other closely related type strains. The scale bar shows 0.02 nucleotide changes per nucleotide position. The phylogenetic tree was created using MrBayes [18] version 3.2.6, sequences were aligned using MAFFT [16, 17]. Numbers at the nodes present the posterior probability

The resulting phylogenetic tree is shown in Fig. 2. Described species of the genera *Oerskovia* and *Cellulomonas* belong to the same family of *Cellulomonadaceae* *.* On the other hand, *Sanguibacter* belongs to the family of *Sanguibacteriaceae* which is defined as a neighboring group to *Cellulomonadaceae* [19]. *Sanguibacter* is the only described genus within the respective family with currently six species [20–24].

## Genome sequencing information

### Genome project history

The genome of *O. enterophila* VJag was sequenced to get insights in the genomic features and the metabolic potential of this strain. Furthermore, no genomes of members of this species were available at the time of writing. A draft sequence is available at NCBI for the species *O. turbata* NRRL B-8019 (JOFV00000000) [25]. The complete genome of *O. enterophila* VJag has a size of 4,535,074 bp and consists of 85 contigs. In this contribution the version LRIE01000000 is described. The genome sequencing and gene annotation was performed by Goettingen Genomics Laboratory (Germany). The sequence can be found under the accession number LRIE00000000. Table 2 shows the project information according to MIGS specification [26].

**Table 2:** Project information

MIGS ID	Property	Term
MIGS 31	Finishing quality	Improved-high-quality draft
		Two genomic libraries: 454 pyrosequencing shotgun library, Illumina paired-end library
MIGS-28	Libraries used	1 kb insert size
MIGS 29	Sequencing platforms	454 GS FLX Titanium, Illumina GAII
MIGS 31.2	Fold coverage	11.46 × 454, 68.28 × Illumina
MIGS 30	Assemblers	MIRA 3.4 and Newbler 2.9
MIGS 32	Gene calling method	Prodigal
	Locus Tag	OJAG
	GenBank ID	LRIE00000000
	GenBank Date of Release	20-APR-2016
	GOLD ID	Gp0050669
	BIOPROJECT	PRJNA309230
MIGS 13	Source Material Identifier	VJag
	Project relevance	Investigation of degradation capabilities of *O. enterophila* VJag

### Growth conditions and genomic DNA preparation

*O. enterophila* VJag was cultivated in 5 ml TSYE-medium (medium 92, DSMZ) at 28 °C overnight in an orbital shaker at 120 rpm for the isolation of genomic DNA. Genomic DNA was isolated using MasterPure Gram positive DNA Purification kit (Epicentre, Madison, WI, USA) according to the manufacturer’s instructions. DNA concentrations and purity were analyzed using the UV-Vis spectrophotometer NanoDrop 2000 (Thermo Fisher Scientific, Waltham, MA, USA). The genomic DNA yield was 2463 ng/μl. The DNA purity was determined using the UV absorbance ratio 260/208 nm and 260/230 nm and revealed ratios of 2.01 and 2.17, respectively.

### Genome sequencing and assembly

A combined approach was used for the whole-genome sequencing of *O. enterophila* VJag using the 454 GS-FLX TitaniumXL system (titanium GS70 chemistry, Roche Life Science, Mannheim, Germany) and the Genome Analyzer II (Illumina, San Diego, CA). According to the manufacturer’s protocols, the shotgun libraries were prepared, which resulted in 97,681 reads for 454 shotgun sequencing (11.46 × coverage) and 4,756,630 112-bp paired end Illumina reads (68.28 × coverage). Illumina reads were trimmed using Trimmomatic 0.32 [27] to remove sequences with quality scores lower than 20 (Illumina 1.9 encoding) and remaining adaptor sequences, respectively. The initial hybrid *de novo* assembly was performed using the MIRA 3.4 [28] and Newbler 2.9 (Roche Life Science, Mannheim, Germany) software. The final assembly resulted in 85 contigs with an average coverage of 79.60, an N50 value of 96,617 bp and an N90 value of 28,097 bp, respectively.

### Genome annotation

The Prodigal software tool [29] was used for automatic gene prediction [29], rRNA and tRNA gene identification was performed using RNAmmer [30] and tRNAscan [31], respectively. The automatic gene-annotation was performed by using the IMG-ER system [32, 33]. The annotation was manually curated using the Swiss-Prot, TrEMBL, and InterPro databases [34].

## Genome properties

**Fig. 3:**
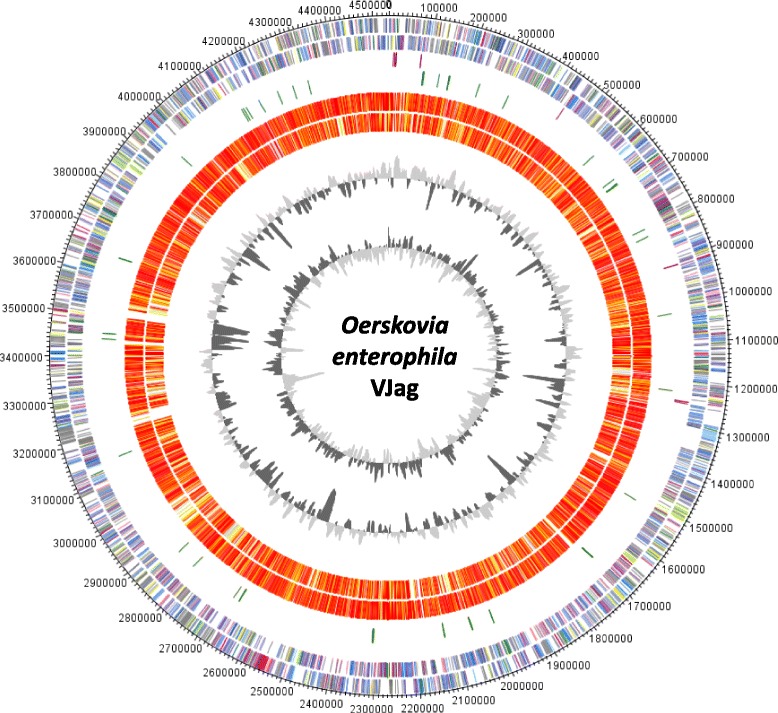
Circular representation of the genome comparison of *O. enterophila* VJag with other *Oerskovia* strains. Outer circles 1 and 2, genes (marked in COG colors) encoded by the leading and the lagging strand of *O. enterophila* VJag; circles 3 and 4, positions of rRNA and tRNA genes, respectively; circle 5, comparison of genes present in *O. enterophila* VJag and *O. enterophila* DFA-19^T^; circle 6, gene comparison of *O. enterophila* VJag and *O. turbata* NRRL B-8019; circle 7 represent the GC content circle 8; represent GC skew. Color code of genome comparison: *grey*: >e^-20^-1; *light yellow*: <e^-50^- > e^-20^; *gold*: <e^-50^- > e^-90^; *light orange*: <e^-90^- > e^-100^; *orange*: <e^-100^- > e^-120^; *red*: <e^-^

The genome of *O. enterophila* VJag is 4,535,074 bp in length and has an average G + C content of 72.4% (Fig. 3). The genome sequence shows 3975 genes in total, 3918 are protein-coding genes, 57 are RNA genes, of which 6 code for rRNA. The remaining genes code for proteins with unknown function or hypothetical proteins. All statistics and properties are listed in Table 3, the number of protein-coding genes associated with general COG functional categories is shown in Table 4.

**Table 3:** Genome statistics

Attribute	Value	% of total
Genome size (bp)	4,535,074	100
DNA coding (bp)	4,016,676	88.57
DNA G + C (bp)	3,283,351	72.40
DNA scaffolds	85	
Total genes	3975	100
Protein coding genes	3918	98.57
RNA genes	57	1.43
Pseudogenes	0	0
Genes in internal clusters	928	23.35
Genes with function prediction	3036	76,38
Genes assigned to COGs	2559	64,38
Genes with Pfam domains	3158	79,45
Genes with signal peptides	332	0,84
Genes with transmembrane helices	1142	28,73
CRISPR repeats	0	0

**Table 4:** Number of genes associated with general COG functional categories

Code	Value	%age	Description
J	199	6.92	Translation, ribosomal structure and biogenesis
A	1	0.03	RNA processing and modification
K	290	10.08	Transcription
L	94	3.27	Replication, recombination and repair
B	1	0.03	Chromatin structure and dynamics
D	27	0.94	Cell cycle control, cell division, chromosome partitioning
V	87	3.02	Defense mechanisms
T	131	4.55	Signal transduction mechanisms
M	134	4.66	Cell wall/membrane/envelope biogenesis
N	11	0.38	Cell motility
U	19	0.66	Intracellular trafficking, secretion, and vesicular transport
O	103	3.58	Posttranslational modification, protein turnover, chaperones
C	145	5.04	Energy production and conversion
G	320	11.12	Carbohydrate transport and metabolism
E	241	8.38	Amino acid transport and metabolism
F	83	2.88	Nucleotide transport and metabolism
H	168	5.84	Coenzyme transport and metabolism
I	109	3.79	Lipid transport and metabolism
P	204	7.09	Inorganic ion transport and metabolism
Q	62	2.16	Secondary metabolites biosynthesis, transport and catabolism
R	283	9.84	General function prediction only
S	156	5.42	Function unknown
-	1416	35.62	Not in COGs

The total is based on the total number of protein coding genes in the genome

A circular representation of the *O. enterophila* VJag genome sequence and comparison to *O. enterophila* DFA-19^T^ [14] and *O. turbata* NRRL B-8019 genome sequences is shown in Fig. 3. For *O. enterophila* VJag the genes encoded by the leading and the lagging strand (outer circles 1 and 2) are marked in COG colors in the artificial chromosome map. The third and fourth circle show the positions of rRNA genes and tRNA genes, respectively. The fifth and sixth circle show comparisons of genes present in the strains *O. enterophila* VJag and *O. enterophila* DFA-19^T^ chromosome as well as *O. enterophila* VJag and *O. turbata* NRRL B-8019 chromosome, respectively. The red colored regions indicate high similarity, whereas yellow colored regions indicate low similarity (see color code, Fig. 3). The two inner most plots represent the GC content and the GC skew (circle 7-8). Furthermore, a pairwise ANI analysis of the VJag strain and type strain *O. enterophila* DFA-19 [14] showed a similarity value of 99.36%, whereas a respective analysis of VJag strain and *O. turbata* NRRL B-8019 resulted in 89.31% similarity.

## Insights from the genome sequence

Because of the close relationship to members of the genus *Cellulomonas*, *O. enterophila* VJag was expected to use cellulose as carbon source. According to the KEGG pathway, genes coding for enzymes probably responsible for the degradation of cellulose to cellobiose and β-D-glucose were found in *O. enterophila* VJag. Cellulose is one of the main components of plant material and is one of the most abundant biopolymers in the environment [35]. Plate assays revealed that *O. enterophila* VJag is able to utilize cellulose [Additional file 3: Figure S1]. The used plates contained CMC as sole carbon source and Congo red to stain CMC. *O. enterophila* VJag hydrolyzed CMC to glucose whereby the Congo red was eluted, the red color got lost and resulted in formation of bright halos around cell spots.

A gene (OJAG_15690) encoding a cellulose 1,4- β -cellobiosidase is present in genome that converts cellulose to 1,4-β-D-glucan. 1,4-β-D-glucan would be further converted to β-D-glucose through the action of a β-glucosidase. The genome sequence of *O. enterophila* VJag comprises 13 genes encoding such β-glucosidases (OJAG_01470, OJAG_39370, OJAG_33570, OJAG_33160, OJAG_31620, OJAG_25090, OJAG_25070, OJAG_16840, OJAG_16640, OJAG_15960, OJAG_15000, OJAG_14990, OJAG_11840).

Furthermore, cellulose can be converted to cellobiose, using endoglucanases (encoded by OJAG_04410; OJAG_07660), and can also be converted to β-D-glucose through the action of a β-glucosidase.

Starch is also ubiquitous in nature as it accumulates in plants as storage compound [36]. The genome sequence of *O. enterophila* VJag harbors genes coding for α-amylases (OJAG_12050; OJAG_09450) and a starch phosphorylase (OJAG_12070). Thus, starch is either converted to glycogen, dextrin, or amylose by *O. enterophila* VJag. Starch or glycogen could also be degraded to trehalose by respective enzymes (glycogen debranching enzyme encoded by OJAG_00790 or OJAG_12120). Subsequently, trehalose would be further converted to β-D-glucose-1-phosphate or D-glucose via an α-trehalose phosphorylase (encoded by OJAG_12210). Dextrin would be converted to α-D-glucose by an oligo-1,6-glucosidase (encoded by OJAG_08510). A plate assay using Jag-MM-agar plates containing starch (2% w/v) as carbon source showed that starch is utilized during growth of *O. enterophila* VJag [Additional file 3: Figure S2]. After incubation, starch was stained using Lugol’s solution and bright halos around cell spots showed starch consumption by *O. enterophila* VJag (see Additional file 3: Figure S2).

Another commonly occurring compound in natural environments besides cellulose and starch is chitin. Chitin is a major structural polymer of the cell walls of fungi and the exoskeletons of invertebrates [37]. Numerous genes which encode enzymes for the degradation of chitin to chitobiose or N-acetylglucosamine were found in the genome sequence of *O. enterophila* VJag (OJAG_26450; OJAG_36940; OJAG_36950; OJAG_36990; OJAG_38030; OJAG_38450; OJAG_38460). Chitobiose could be converted to N-acetylglucosamine or N-acetylglucosamine-1-phosphate by hexosaminidases and β-N-acetylhexosaminidase (encoded by OJAG_07390; OJAG_13640; OJAG_13650; OJAG_33360; OJAG_35500; OJAG_09950; OJAG_30030; OJAG_15920). Furthermore, one gene (OJAG_13250) was found that encodes a glucosamine-1-phosphate N-acetyltransferase, which converts N-acetylglucosamine-1-phosphate to UDP-acetylglucosamine. This intermediate would be further transformed to N-acetylglucosamine enopyruvate by an UDP-N-acetylglucosamine-1-carboxyvinyltransferase (encoded by OJAG_15040; OJAG_22690). N-acetylglucosamine enopyruvate can subsequently be converted to N-acetylmuramic acid via an UDP-N-acetylmuramate dehydrogenase (encoded by OJAG_01210). N-acetylmuramic acid would be metabolized through the peptidoglycan biosynthesis pathway or the D-glutamin and D-glutamate metabolism (OJAG_14230; _ OJAG 14240).

Additionally, genes encoding enzymes for xylose degradation were found in the *O. enterophila* VJag genome sequence. D-xylose could be converted to D-xylulose by a xylose isomerase (encoded by OJAG_26770). Furthermore, D-xylulose would be phosphorylated to D-xylulose-5-phosphate via a xylulokinase (OJAG_26780). D-xylulose-5-phosphate would be converted to D-ribulose-5-phosphate by a ribulose-5-phosphate 3-epimerase (OJAG_00210), and then metabolized via the pentose phosphate pathway, or D-xylulose-5-phosphate would be converted to L-ribulose -5-posphate via a L-ribulose-5-phosphate 4-epimerase (OJAG_27380). This also fits into the overall picture since xylose is a main part of hemicellulose and makes up a part of plant materials [38].

## Conclusions

The genome of *O. enterophila* VJag, which was isolated from forest soil, is described. Furthermore, the phylogenetic and phenotypic characteristics of the isolated strain are presented. It has been shown that the isolate belongs to the family of *Cellulomonadaceae*. Scanning electron micrographs confirmed the variable phenotype in exponential or stationary growth phase. Genome sequences analysis revealed that *O. enterophila* VJag has the genetic properties to degrade compounds typically abundant in forest soils. Plate assays showed that the isolated strain is able to use starch and cellulose as sole carbon and energy source. The genome sequence of *O. enterophila* VJag has been deposited at DDBJ/EMBL/GenBank and can be found under the accession number LRIE00000000. The version described in this paper is version LRIE01000000.

## Additional files

Additional file 1: Enrichment, isolation and selection of bacterial strains; identification of isolated strains (S1). (DOCX 15 kb)

Additional file 2: Detailed composition of Jag-MM agar and silica plates (S2). (DOCX 17 kb)

Additional file 3: Figure S1. Jag-MM-silica plates with CMC and Congo red; **Figure S2.** Jag-MM-agar plates with starch. (ZIP 964 kb)

## Abbreviation

CMC: Carboxymethyl cellulose
