# Supplementary material for: Genome sequencing and description of Oerskovia enterophila VJag, an agar- and cellulose-degrading bacterium
Source: Stand Genomic Sci. 2017 May 4;12:30. doi: 10.1186/s40793-017-0244-4 (PMC5418683; doi:10.1186/s40793-017-0244-4)
Supplement: Supplementary file 1 — Enrichment, isolation and selection of bacterial strains; identification of isolated strains (S1). (DOCX 15 kb) [file 40793_2017_244_MOESM1_ESM.docx]

## Enrichment, isolation and selection of bacterial strains

Soil samples were collected from the botanical garden of Universität Ulm, Ulm, Germany (N 48° 25.331ˈ; E 09° 57.553ˈ). The isolation procedure was as follows: 1 g of soil sample was mixed in 9 ml of sterile 0.9 % saline (9 g NaCl in 1000 ml demin. water), which resulted in a dilution of 1:10. The solution was mixed thoroughly for at least 1 minute. The supernatant was again diluted with sterile 0.9 % saline after an incubation of 1 to 5 minutes at room temperature (RT). 100 μl of the following dilutions were plated on Jag-minimal-agar-plates: 1:10, 1:100, 1:250 and 1:1000 (S2) to obtain bacteria, which are able to use agar as sole carbon and energy source. Single colonies, grown on these plates, were streaked on fresh Jag-minimal-agar-plates to get pure cultures. Each of them were again incubated at 28 °C for 7-12 days. This procedure was repeated three times. The enrichment procedure resulted in 28 strains, of which 4 were identified as *O. enterophila*. *O. enterophila* VJag was selected for further characterization.

**Identification of isolated strains**

In total 4 strains were selected for identification by subsequent 16S RNA gene analysis. Therefore, genomic DNA of the isolates was prepared and a polymerase chain reaction performed using the primers 16S-27F (5′-ATAAGCTTGGATCCAGAGTTTGATCCTGGCTCAG-3′) and 16S-1492r (5′-ACTCGAGGATATCGGTTACCTTGTTACGACTT-3′) [1]. The PCR mixture contained FailSafe™ PCR PreMix (epicentre^®^, Maison, [USA](https://www.google.com/maps/place/United+States)), 20 μl; ReproFast polymerase (Genaxxon Bioscience GmbH, [Ulm, Germany](https://www.google.com/maps/place/Ulm,+Baden-Wurttemberg,+Germany)), 0.5 μl and 1 μl of each primer in a final volume of 50 μl. The amplification of the 16S rRNA gene fragments was performed using a thermal cycler (S1000™, BioRad, [Munich, Germany](https://www.google.com/maps/place/Munich,+Bayern,+Germany)). The first step of the PCR was an initial denaturation for 5 min at 95 °C, followed by the DNA amplification in 32 cycles at 95 °C for 45 seconds, 55 °C for 45 seconds and 72 °C for 1.5 minutes. This amplification was followed by a final elongation at 72 °C for 10 minutes. The obtained PCR fragments were purified using “DNA Clean & Concentrator-5-Kit™“ (Hiss Diagnostics GmbH; Freiburg, [Germany](https://www.google.com/maps/place/Germany)) and sequenced by GATC Biotech AG ([Konstanz, Germany](https://www.google.com/maps/place/Konstanz,+Baden-Wurttemberg,+Germany)). The received 16S RNA gene sequences were then analyzed using the “Nucleotide BLAST” software from NCBI [2].

**References**

1. Lane DJ. 16S/23S rRNA sequencing. In: Nucleic acid techniques in bacteria systematics. Stackebrandt E, Goodfellow M, editors. New York: Wiley and Sons; 1991. p.115-175.
2. Johnson M, Zaretskaya I, Raytselis Y, Merezhunk Y, McGinnis S, Madden TL: NCBI BLAST: a better web interface. Nucleic Acids Res. 2008; doi:10.1093/nar/gkn201.
