# Supplementary material for: Genome sequencing and description of Oerskovia enterophila VJag, an agar- and cellulose-degrading bacterium
Source: Stand Genomic Sci. 2017 May 4;12:30. doi: 10.1186/s40793-017-0244-4 (PMC5418683; doi:10.1186/s40793-017-0244-4)
Supplement: Supplementary file 2 — Detailed composition of Jag-MM agar and silica plates (S2). (DOCX 17 kb) [file 40793_2017_244_MOESM2_ESM.docx]

**Detailed composition of Jag-MM agar plates and Jag-MM silica plates**

**Jag-minimal medium (Jag-MM)**

(NH_4_)_2_SO_4_ 0.5 g 3.7 mM

KH_2_PO_4_ 1.36 g 10 mM

K_2_HPO_4_×2 H_2_O 3.2 g 18.37 mM

Vitamin solution No. 6 10 ml 1 % (w/v)

Trace element solution 10 ml 1 % (w/v)

Agar 15 g 1.5 % (w/v)

H_2_O demin. ad 1000 ml

**Vitamin solution No. 6** [1]

Biotin 2 mg 8.1 µM

Folic acid 2 mg 4.5 µM

Vitamin B6 10 mg 60 µM

Riboflavin 5 mg 13 µM

Vitamin B1 5 mg 17 µM

Nicotinamid 5 mg 41 µM

Panthotenic acid 5 mg 23 µM

Vitamin B12 0.1 mg 0.063 µM

Aminobenzoic acid 5 mg 36 µM

H_2_O demin. ad 1000 ml

**Trace element solution – „Modified Balch’s Trace Element Solution“** [1, mod]

Nitrilotriacetic acid 1.5 g 7.8 mM

MgSO_4_×7 H_2_O 3 g 12.17 mM

NaCl 1 g 17.1 mM

MnSO_4_×H_2_O 0.5 g 2.9 mM

FeSO_4_×7 H_2_O 0.1 g 0.36 mM

CoCl_2_×6 H_2_O 0.1 g 0.42 mM

CaCl_2_ 0.1 g 0.9 mM

ZnSO_4_×7 H_2_O 0.1 g 0.35 mM

CuSO_4_×5 H_2_O 0.01 g 0.04 mM

AlK(SO_4_)_2_×12 H_2_O 0.01 g 0.02 mM

H_3_BO_3_ 0.01 g 0.16 mM

Na_2_MoO_4_×2 H_2_O 0.01 g 0.04 mM

NiSO_4_×6 H_2_O 0.03 g 0.114 mM

Na_2_SeO_3_×5 H_2_O 0.02 g 0.12 mM

Na_2_WO_4_×2 H_2_O 0.02 g 0.06 mM

H_2_O demin. ad 1000 ml

The vitamin solution No. 6 and the trace element solution were added to the medium after the autoclaving. Both solutions were sterile filtrated before.

For the preparation of silica media plates, the following substances were added:

**20 % (v/v) phosphoric acid [2]**

Phosphoric acid, 85 % (v/v) 200 ml 20 % (w/v)

H_2_O demin. ad 1000 ml

**Potassium silicate [2]**

KOH 70 g 7 % (w/v)

Silica 100 g 10 % (w/v)

H_2_O demin. ad 1000 ml

For solving the silica completely in the potassium hydroxide, the solution has to be heated to approx. 60-70 °C for at least one hour. Afterwards, the solution was autoclaved at 121 °C for 15 minutes.

**2x TY medium [3]**

Tryptone 16 g 1.6 % (w/v)

Yeast extract 10 g 1 % (w/v)

NaCl 5 g 0,5 % (w/v)

H_2_O demin. ad 1000 ml

**LB-medium [3]**

Tryptone 10 g 1 % (w/v)

Yeast extract 5 g 0.5 % (w/v)

NaCl 10 g 1 % (w/v)

H_2_O demin. ad 1000 ml

**References**

1. Staley JT. *Prosthecomicrobium* and *Ancalomicrobium*: new prosthecate freshwater bacteria. J Bacteriol. 1968; 95:1921-1942.

**2. Funk H, Krulwich T. Preparation of clear silica gels that can be streaked.** J Bacteriol. **1964;** 88:1200-1201.

3. Green MR, Sambrook J. Reagents and buffers. In: Inglis J, Boyle A, Gann A, editors. Molecular cloning. Cold Spring Harbor Laboratory Press; 2012. p. 1811-1842.
