# Supplementary material for: Genome sequencing and description of Oerskovia enterophila VJag, an agar- and cellulose-degrading bacterium
Source: Stand Genomic Sci. 2017 May 4;12:30. doi: 10.1186/s40793-017-0244-4 (PMC5418683; doi:10.1186/s40793-017-0244-4)
Supplement: Supplementary file 3 — Jag-MM-silica plates with CMC and Congo red; Figure S2. Jag-MM-agar plates with starch. (ZIP 964 kb) [file 40793_2017_244_MOESM3_ESM.zip › Additional File 3.docx]

**Fig. 1S** Jag-MM –silica-plates with 1 % CMC, stained with Congo red. Bright halos around cell spots indicate cellulose degradation. **A:** *O. enterophila* VJag, **B:** *C. uda* CG1**, C:** *E. coli* DH5α, **D**: 0.9 % saline.

Cells of *O. enterophila* VJag were tested regarding their ability to utilize carboxymethyl cellulose (CMC). Therefore, Jag-MM-silica-plates were used, that contained Congo Red stained CMC (1 % w/v). *Cellulomonas uda* CG1 cells are able to degraded cellulose [1,2] and were used as positive control, whereas *E. coli* DH5α [3] cells served as negative control in this assay. Overnight culture of *O. enterophila* VJag, *C. uda* CG1, and *E. coli* DH5α were cultivated either in 5 ml TSYE-medium, LB-medium, or 2x TY-medium, respectively. After incubation cells were washed two times using 0.9 % NaCl-solution and finally solved in 500 µl of 0.9 % NaCl-solution. 10 µl of each cell culture were spotted onto Jag-MM-silica-plates (1 % CMC) and incubated at 30 °C for at least 6 days.

**Fig. 2S** Jag-MM-agar-plates with 2 % starch, lower row stained with lugol’s solution. **A:** *O. enterophila* VJag, **B:** *C. uda* CG1, **C:** *E. coli* DH5α, **D:** 0.9 % saline.

Starch degradation of O. enterophila VJag was analysed using Jag-MM-agar plates supplemented with 2 % (w/v) starch. Cells of Cellulomonas uda CG1 were used as positive control since they also capable to degraded starch [4]. E. coli DH5α [5] cells were used as negative control in this assay since they are not able to degrade starch. Pre-cultivation, washing, and spotting of cells onto Jag-MM-agar plates was performed as described before (see CMC-assay). After incubation of cells at least days for 6 at 30 °C, starch was stained with Lugol’s solution [6]. Bright halos around cell spots showed starch hydrolysis (Fig S2).

1. Stoppok W, Rapp P, Wagner F. Formation, location and regulation of endo-1,4-β-glucanases and β-glucosidasesfrom *Cellulomonas uda*. Appl Environ Microbiol. 1982;44:44-53.
2. Poulsen HV, Willink FW, Ingvorsen K. Aerobic and anaerobic cellulase production by *Cellulomonas uda*. Arch Microbiol. 2016; doi:10.1007/s00203-016-1230-8.
3. Gao D, Luan Y, Wang Q, Liang Q, Qi Q. Construction of cellulose-utilizing *Escherichia coli* based on a secretable cellulase. Microb Cell Fact. 2015; doi:10.1186/s12934-015-0349-7.
4. Kumar NN, Bhide A, Gokhale DV, Deobagkar DN. Production of extracellular amylase by *Cellulomonas* sp NCIM 2353. Biotechnol Appl Biochem. 1995;22:345-353.
5. Rosales-Colunga LM, Martínez-Antonio A. Engineering *Escherichia coli* K12 MG1655 to use starch. Microb Cell Fact. 2014; doi:10.1186/1475-2859-13-74.
6. Seibold G, Auchter M, Berens S, Kalinowski J, Eikmanns BJ. Utilization of soluble starch by a recombinant *Corynebacterium glutamicum* strain: growth and lysine production. J Bacteriol. 2006;124:381-391.
