# Supplementary figures and images for: Genome sequencing and description of Oerskovia enterophila VJag, an agar- and cellulose-degrading bacterium
Source: Stand Genomic Sci. 2017 May 4;12:30. doi: 10.1186/s40793-017-0244-4 (PMC5418683; doi:10.1186/s40793-017-0244-4)

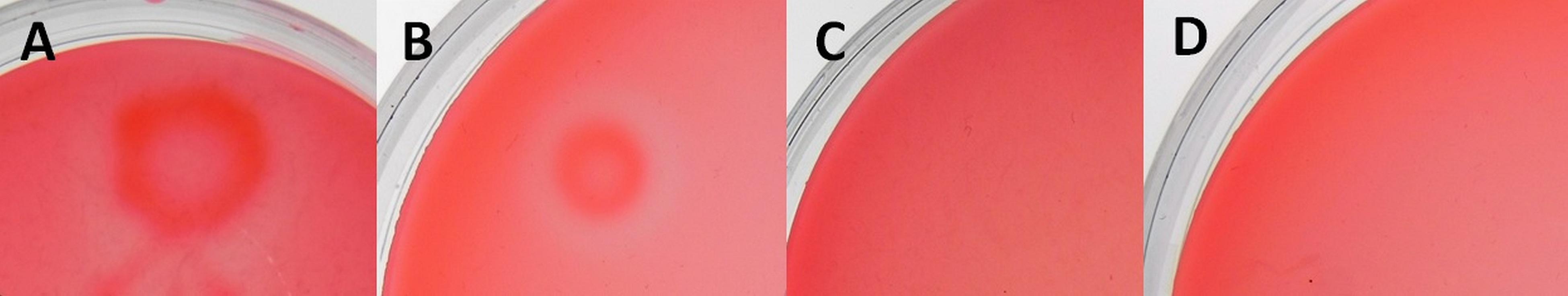

Supplement: Supplementary file 3 — Jag-MM-silica plates with CMC and Congo red; Figure S2. Jag-MM-agar plates with starch. (ZIP 964 kb) [file 40793_2017_244_MOESM3_ESM.zip › Fig. 1S.jpg]

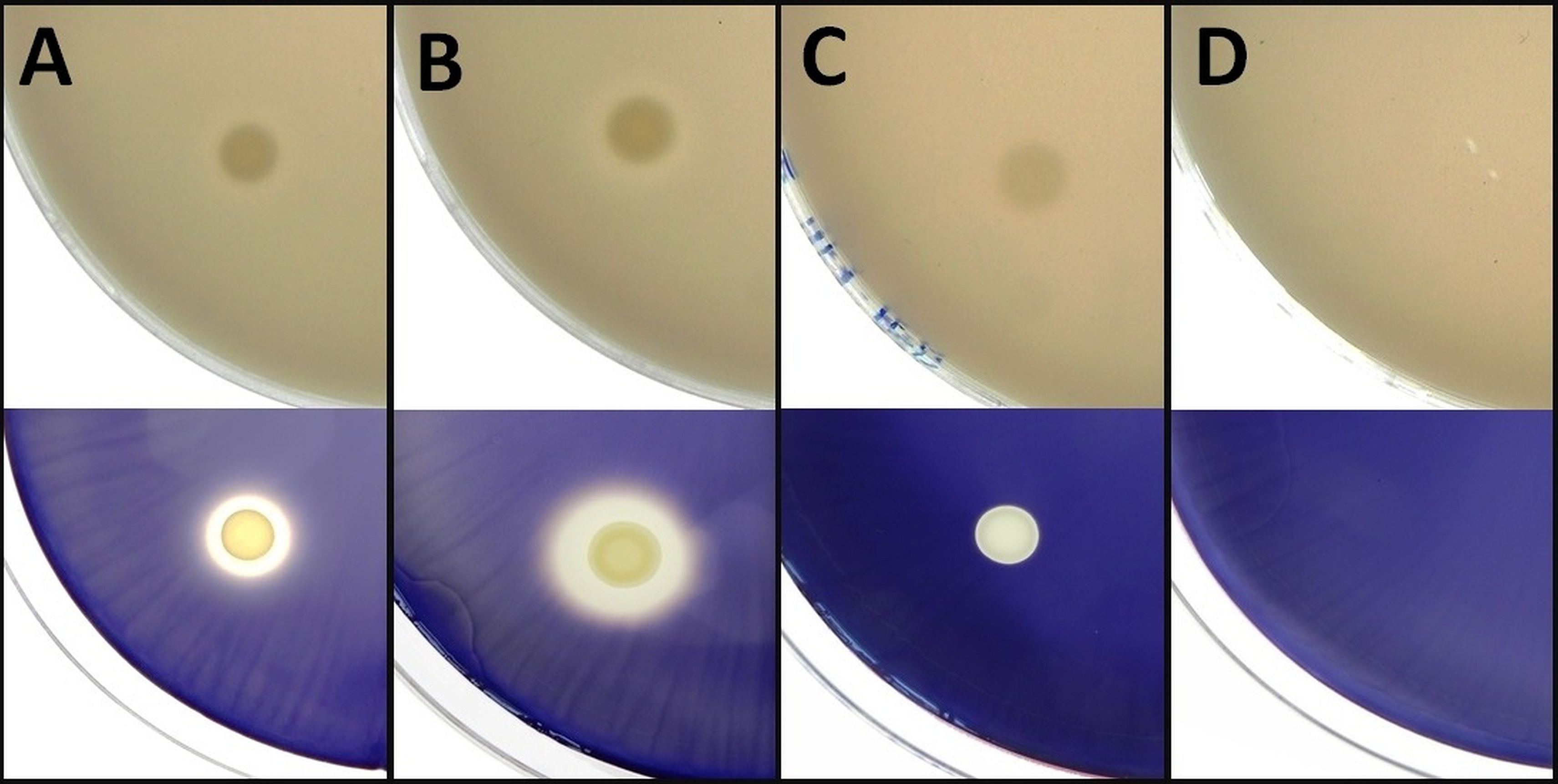

Supplement: Supplementary file 3 — Jag-MM-silica plates with CMC and Congo red; Figure S2. Jag-MM-agar plates with starch. (ZIP 964 kb) [file 40793_2017_244_MOESM3_ESM.zip › Fig. 2S.jpg]
